# Functions of Papillomavirus E8^E2 Proteins in Tissue Culture and In Vivo

**DOI:** 10.3390/v14050953

**Published:** 2022-05-02

**Authors:** Franziska Kuehner, Frank Stubenrauch

**Affiliations:** Institute for Medical Virology and Epidemiology of Viral Diseases, University Hospital Tuebingen, Elfriede-Aulhorn-Str. 6, 72076 Tuebingen, Germany; franziska.kuehner@med.uni-tuebingen.de

**Keywords:** papillomavirus, HPV, E2, E8^E2, MmuPV1, NCoR, SMRT, HDAC3

## Abstract

Papillomaviruses (PV) replicate in undifferentiated keratinocytes at low levels and to high levels in differentiated cells. The restricted replication in undifferentiated cells is mainly due to the expression of the conserved viral E8^E2 repressor protein, a fusion protein consisting of E8 and the hinge, DNA-binding, and dimerization domain of E2. E8^E2 binds to viral genomes and represses viral transcription and genome replication by recruiting cellular NCoR/SMRT-HDAC3 corepressor complexes. Tissue culture experiments have revealed that E8^E2 modulates long-term maintenance of extrachromosomal genomes, productive replication, and immortalization properties in a virus type-dependent manner. Furthermore, in vivo experiments have indicated that Mus musculus PV1 E8^E2 is required for tumor formation in immune-deficient mice. In summary, E8^E2 is a crucial inhibitor whose levels might determine the outcome of PV infections.

## 1. Introduction

**Figure 1:**
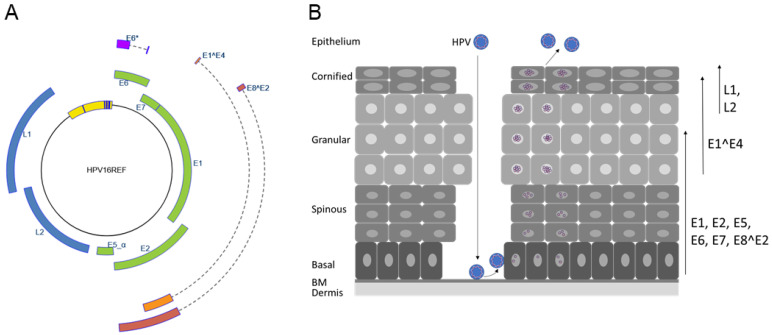
The HPV genome and life cycle. (**A**) The human papillomavirus 16 genome structure. Open reading frames *E1*, *E2*, *E5*, *E6*, *E7*, *L1,* and *L2* are indicated. Spliced mRNAs (indicated by dashed lines) are used to generate the E6*, E1^E4, and E8^E2 proteins. The upstream regulatory region, which is located between the *L1* and *E6* genes harboring the origin of replication and transcription control elements, is depicted in yellow, and blue bars indicate conserved E1 and E2 binding sites. The illustration was taken from https://pave.niaid.nih.gov/#explore/reference_genomes/human_genomes (accessed on 1 January 2020) [1]. (**B**) Schematic representation of the stratified epithelium and the HPV life cycle. HPV virions infect basal cells adjacent to the basal membrane (BM) via microlesions in the epithelial barrier. The viral life cycle is tied to differentiation and ends with the production of progeny virus that are released from terminally differentiated cells. Arrows on the right indicate differentiation-dependent expression of viral proteins. After introduction of viral genomes into undifferentiated keratinocytes, the viral E5, E6, and E7 oncoproteins as well as the replication modulatory E1, E2, and E8^E2 proteins are expressed. Expression of E1^E4 coincides with viral genome amplification in the spinous and granular layers. The expression of L1 and L2 in the terminally differentiated cells of the upper epithelium leads to the packaging of viral genomes, virion synthesis, and the release of virions.

Papillomaviruses (PV) are non-enveloped, double-stranded DNA viruses with currently more than 440 different sequenced genotypes, of which 220 infect humans [1]. Infections with human papillomaviruses (HPV) can cause different kinds of warts or neoplasias on cutaneous or mucosal epithelia. Persistent infections with high-risk (HR) HPV types 16, 18, 31, 33, 35, 39, 45, 51, 52, 56, 58, and 59 are the major risk factor for the development of cervical and other ano-genital cancers and a fraction of cancers of the oropharynx [2]. On the other hand, HPV from the genus beta have been implicated in the development of cutaneous squamous cell cancer in patients with the rare genodermatosis epidermodysplasia verruciformis and in organ transplant recipients [3,4]. Papillomaviruses infect keratinocytes in the basal layer of cutaneous or mucosal skin. However, virus capsid protein expression and amplification of viral genomes to high levels takes only place in the upper layers of the epithelium (Figure 1). Available data do not support the idea that viral proteins other than the L1 and L2 capsid proteins are part of infectious virions. Consistent with this, viral early promoters have robust basal activity in keratinocytes in the absence of viral gene products [5]. This is due to the presence of binding sites for different host transcription factors in the upstream regulatory region (URR) located between the L1 and E6 genes and also in the coding region for the viral early genes (Figure 1A, [5]). Initial transcription results in the expression of early viral proteins, which modulate genome replication (E1, E2, E8^E2) and modulate viral gene expression (E2, E8^E2) and host cell pathways (E5, E6, E7) (Figure 1A). Upon differentiation, genomes are further amplified, and additional viral promoters are activated. Furthermore, polyadenylation site usage and splicing patterns of viral transcripts change, resulting in the differentiation-dependent expression of the late E1^E4, L1, and L2 proteins, which leads to the synthesis of infectious virions (Figure 1B). Thus, viral gene expression and replication are tightly controlled by host cell and viral proteins and the differentiation state of the infected cell.

## 2. The E2 Protein

Early after introduction of viral genomes into undifferentiated keratinocytes, the viral E5, E6, and E7 oncoproteins as well as the E1, E2, and E8^E2 replication proteins are expressed (Figure 1). E1, E2, and E8^E2 are highly conserved, sequence-specific DNA binding proteins. The E1 protein recognizes the viral origin of replication located in the URR, and its helicase activity serves to unwind DNA, which is then replicated by the recruitment of the cellular DNA replication machinery [6]. E1 only binds tightly to the viral origin in a complex with the viral E2 protein, which is mediated by the conserved ~200 aa N-terminal domain of E2 [6,7]. The E2 C-terminal domain of ~100 aa is responsible for the specific recognition of DNA sequences (ACCN_6_GGT; E2 binding sites (E2BS)) and also for the dimerization of E2 proteins [7]. Consistent with its function as a helicase loader, E2 is essential for the replication of PV genomes [8,9,10]. In addition, E2 can also activate transcription, and this is crucial for bovine papillomavirus 1 (BPV1) gene expression [7]. In contrast, transcriptional activation by HPV31 E2 is not necessary for viral genome replication in undifferentiated keratinocytes but may contribute to the expression of late viral genes in differentiated cells [11,12,13]. Overexpression of E2 represses transcription from the major early promoter of HR-HPV, which has been suggested to be an important control mechanism to limit expression of the viral E6 and E7 oncoproteins. However, only mutations in the DNA-binding domain (DBD) but not in the N-terminal domain of E2 increased viral early gene expression of HPV16 genomes strengthening the concept that E8^E2 repressor proteins that share the C-terminal but not the N-terminal domain with E2 are responsible for the repression of transcription [14]. Thus, the contribution of transcriptional repression by E2 to the replication of HR-HPV remains controversial.

## 3. E2 Repressor Proteins

### 3.1. Transcripts for PV Repressor Proteins

Early studies with bovine papillomavirus 1 (BPV1) indicated that additional proteins are made from the E2 gene that were labeled E2TR (or E2C) and E8/E2 [15,16,17]. E2TR is an N-terminally truncated protein that is derived from an RNA initiating at an E2 internal promoter [17]. The E8/E2 mRNA is derived from a promoter in E1 and is spliced from nt. 1235 to nt. 3225, resulting in E8 residues 1–11 fused to E2 residues 207–410 [15]. Transcript analyses indicate that animal PV (Macaca fascicularis (Mf) PV1, MfPV5, MfPV8, Mus musculus (Mmu) PV1, Mastomys natalensis (Mn) PV1, Sylvilagus floridanus (Sf) PV1 (or cottontail rabbit) CRPV) as well as HPV1, 5, 8, 11, 16, 18, 31, 33, and 49 express a spliced RNA homologous to the BPV1 E8/E2 mRNA (Figure 1) [8,14,18,19,20,21,22,23,24,25,26,27,28,29,30]. Bioinformatic analyses suggest that the potential to generate E8^E2 transcripts and the corresponding fusion proteins is highly conserved, as E8 exons can be found in more than 300 mammalian PV genomes, including all HPV types in the alpha, beta, gamma, and mu-genera [19,31]. The HPV E8^E2 mRNA is generated from a separate promoter within the E1 gene with transcriptional start sites located 70–150 nt upstream of the E8 ATG start codon [8,14,20,29,32,33,34,35]. Transcript analyses of HPV16 suggest that the main function of this promoter is to drive E8^E2 expression [34]. Up to now, only the HPV16 E8 promoter has been analyzed in detail, which revealed that it displays basal activity in HPV-negative keratinocytes and that this is dependent upon conserved DNA sequences close to the transcription start site that are bound by cellular proteins [34].

### 3.2. Phenotypes of E8^E2 Repressor Knock-Out Genomes

#### 3.2.1. Tissue Culture Experiments

The analysis of BPV1 E2TR knock-out (ko) genomes in murine C127 cells revealed that E2TR limits BPV1 replication [17,36]. In contrast, a E8/E2 ko had no obvious phenotype [37]. Remarkably, BPV1 E8/E2 ko/E2TR ko genomes displayed reduced replication activity and transformation efficiency [37]. Sequence analysis of BPV1 genomes isolated from E8/E2 ko/E2TR ko transformed cells revealed frequent reversions of the inactivating mutation in E8 but not in E2TR [38], indicating that E8/E2 is important for cell transformation. HPV1, 5, 8, 11, 16, 18, 31, and 49 E8^E2 ko genomes replicate to much higher levels than wild-type (wt) genomes in short-term assays in immortalized human keratinocytes (HPV16, 31), normal human keratinocytes (HPV1, 8, 16, 31, 49), or the U2OS osteosarcoma cell line (HPV5, 11, 18) [8,14,20,23,27,39,40,41,42]. Furthermore, MmuPV1 E8^E2 ko genomes display greatly increased viral gene expression in cultured murine keratinocytes similar to HPV E8^E2 ko in human keratinocytes suggesting that E8^E2 function is conserved among human and animal PVs [43]. Evolutionary conservation, viral mutants and complementation assays strongly indicate that the E8^E2 fusion protein is responsible for the phenotypes of PV E8^E2 mutants: (1) Disruption of the E8 exon splice donor site, the E8 ATG, or translation termination linker (TTL) mutants in E8 result in increased genome replication [14], arguing that the spliced E8^E2 product is responsible. (2) TTL mutants in E8 or E2 downstream of the splice acceptor used to generate the E8^E2 mRNA but not in E2 upstream of the splice acceptor increase activity of the viral major early promoter P97 [14], which also shows that E8^E2 derived from the spliced mRNA is the main transcriptional repressor. (3) Complementation assays with HPV16 genomes also support the idea that E8^E2 acts in trans to limit genome replication [14]. (4) E8^E2 expression plasmids inhibit replication of HPV E8^E2 ko genomes in a concentration-dependent manner [8,14]. In summary, the E8^E2 fusion protein is a crucial inhibitor of HPV replication and gene expression.

**Figure 2:**
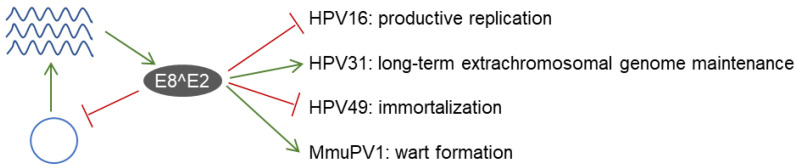
E8^E2 is a conserved repressor of viral replication and gene expression. E8^E2 is expressed from PV genomes and then limits genome replication (blue circle represents the viral genome) and gene expression (viral mRNA is shown as wavy blue lines). E8^E2 expression is required for long-term extrachromosomal maintenance of HPV31 and wart formation by MmuPV1 in vivo. In contrast, E8^E2 restricts productive replication of HPV16 and prevents immortalization of HPV49.

While increased replication and gene expression in short-term assays is a highly conserved phenotype for PV E8^E2 ko genomes, long-term cultivation revealed unexpected differences (Figure 2). HPV16 E8^E2 ko genomes are stably maintained with elevated copy numbers as extrachromosomal elements, revealing that the short-term phenotype can be long-term maintained [14,41]. In contrast, HPV31 E8^E2 ko genomes cannot be stably maintained as episomes [14,27]. The beta-HPV49 wt genome is not maintained in human keratinocytes despite encoding and transcribing E6 and E7 oncoproteins with immortalizing activity, whereas an HPV49 E8^E2 ko resulted in immortalized keratinocytes maintaining high copy number extrachromosomal plasmids [23,44]. This indicates that E8^E2 controls immortalization by inhibiting E6 and E7 transcript levels and limiting genome replication [23]. E8^E2 restricts viral replication in undifferentiated cells in culture, which are thought to mimic infected keratinocytes in the basal layer of epithelia. Thus, E8^E2 could be involved in the switch from the non-productive to the productive PV replication cycle in differentiating keratinocytes. Surprisingly, HPV16 E8^E2 ko genomes also displayed higher levels of viral genomes, transcripts, and the late viral proteins in differentiated cells than the wt [41]. This indicated that HPV16 E8^E2 also limits productive replication in differentiated cells (Figure 2), This could be an immuno-evasive strategy of HPV16 in order to limit the amounts of immunogenic viral proteins such as L1.

#### 3.2.2. Phenotypes in Animal Models

CRPV expresses an E8^E2 (originally designated as E9^E2C) transcript comparable to HPV, BPV1, and MmuPV1 [21]. Surprisingly, E8^E2 ko genomes lacked an in vivo phenotype in domestic rabbits, as they did not differ from wt genomes in tumor induction rates, tumor sizes, viral transcription, or viral copy numbers [21]. However, no evidence was provided that the lack of E8^E2 expression really had an impact on CRPV genome replication or gene expression in cultured rabbit keratinocytes. It is thus possible that E8^E2 is not expressed in sufficient amounts in domestic rabbit keratinocytes, which would explain the lack of a phenotype in vivo. In contrast, studies with MmuPV1 revealed that the knock-out of E8^E2 increases early and late viral gene transcription in murine keratinocytes similar to HPV E8^E2 ko in human keratinocytes [43]. Remarkably, MmuPV1 E8^E2 ko genomes neither induced tail warts nor were able to maintain long-term gene expression in the vaginal tract of immunodeficient nude mice (Figure 2) [43]. This suggests that the primary function of MmuPV1 E8^E2 in vivo is not the limitation of viral gene expression to avoid immune detection but hints at an important requirement for E8^E2 for tumor formation in the absence of cellular immunity.

### 3.3. Mechanisms of E2 Repressor Protein Activities

The repressive effect of E2TR on BPV1 replication is caused by interfering with both E2’s transactivation and replication functions [17,45]. In contrast, heterodimerization between E2 and E2TR does not play a major role, as E2/E2TR heterodimers or E2 single-chain heterodimers consisting of the E2 DBD and fused to full-length E2 recruit the E1 helicase, support viral DNA replication, and also activate E2-dependent transcription [45,46,47]. Inhibition of viral replication and gene expression by E2TR homodimers is therefore mainly due to binding site competition with E2 homodimers and E2/E2TR heterodimers at E2BS [45].

BPV1 E8/E2 inhibits focus formation and activation of transcription by E2 [15,37]. BPV1 E8/E2 also represses constitutive promoter activity suggestive of E2-independent activities [15,48]. The mechanism of repression by BPV1 E8/E2 has not been investigated, but the specific reconstitution of E8/E2 expression in transformed cells derived from E2TR/E8/E2 ko genomes makes it likely that E8/E2 has different activities than E2TR.

**Figure 3:**
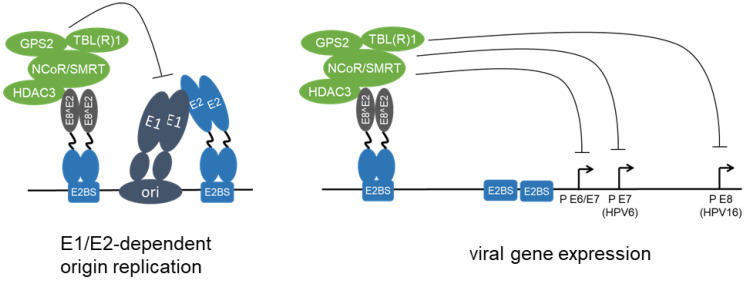
HPV E8^E2 proteins repress viral replication and transcription. HPV E8^E2 proteins bind to E2BS via the DNA binding domain within the C-terminal part shared by full-length E2 and E8^E2. The E8 domain recruits NCoR/SMRT corepressor complexes composed of GPS2, HDAC3, NCoR, SMRT, TBL1, and TBLR1, and this inhibits both E1/E2-dependent replication of the viral origin and the transcription from different viral promoters.

The general increase in viral transcription upon E8^E2 inactivation suggests that E8^E2 might regulate all viral promoters [23,41]. In line with this, analyses of reporter constructs have revealed that HR-HPV major early promoters, the HPV6 E7 promoter, the HPV16 E8 promoter, and MmuPV1 promoters, can be efficiently repressed by E8^E2 [27,34,43,49,50]. E8^E2 is an efficient repressor of E6 and E7 transcription, and this is relevant for cellular immortalization by HPV49 as well as the growth of HPV18-positive HeLa cells [19,23,50]. In contrast to E2, transcriptional repression by E8^E2 acts over a distance of more than 1 kbp (Figure 3) [34,51]. Furthermore, E8^E2 inhibits the E1/E2-dependent replication of the viral origin (Figure 3) [27,39,41,42,43]. Thus, increased replication of E8^E2 ko genomes is most likely due to a combined effect of increased viral transcription and de-repressed E1/E2-dependent replication.

Transcriptional repression requires one E2BS and the DBD of E8^E2 [14,50,51], consistent with the idea that E8^E2 acts mainly as a sequence-specific DNA binding transcription factor. The high conservation of the E8 part suggested that it contributes to E8^E2 repressive activities. In line with that, the deletion of E8 from E8^E2 proteins attenuates both inhibition of transcription and of E1/E2-dependent replication [21,39,43,51]. Highly conserved residues K5, W6, and K7 in alpha-PV or K2, L3, and K4 in beta- and mu-PV are required for both the repression of transcription and E1/E2-dependent origin replication [39,41,42,50,51]. HPV16 and 31 E8 KWK mt genomes replicate to high levels, confirming that the E8 part is crucial for E8^E2’s activities in infected cells [41,42]. Replacing the DBD/dimerization domain of E8^E2 with a heterologous DBD/dimerization domain resulted in a DNA binding site dependent transcription repressor and inhibitor of E1/E2-dependent replication in a E8 KWK motif-dependent manner [42,52]. This revealed that the E8 part has repression activity on its own and suggested that the inhibition of E1/E2 dependent-origin replication does not mainly result from binding site competition between E2 and E8^E2.

The observation that the N-terminus of HPV31 E8^E2 represents a transferable transcriptional repression domain suggested that it might recruit host cell transcription factors. In line with that, class I histone deacetylases (HDAC) 1, 2, and 3 were identified as E8 domain-dependent interactors in a candidate approach [52]. Affinity-purification/mass spectrometry approaches confirmed HDAC3 and additionally identified GPS2 (G protein pathway suppressor 2), NCoR (Nuclear receptor corepressor), SMRT/NCoR2 (Silencing mediator of retinoic acid and thyroid hormone receptor/Nuclear receptor corepressor 2), TBL1 (Transducin-beta-like protein 1), and TBLR1 (TBL1-related protein 1) as high-confidence interactors for HPV16 and 31 E8^E2 proteins (Figure 3, [39,53]). These proteins are known to form stable NCoR/SMRT corepressor complexes, which have been shown to mediate the transcriptional repression activity of several cellular transcription factors, such as c-myc, estrogen receptor, MeCP2, peroxisome proliferator activated receptors, retinoic acid receptor, Rev-erbα, and thyroid hormone receptor [54,55,56,57,58,59,60,61]. NCoR and SMRT are highly homologous proteins that serve as scaffolding proteins by interacting with GPS2, HDAC3, TBL1, and TBLR1 [62]. TBL1 and TBLR1 are also homologous proteins that form homotetramers or heterotetramers [62]. The enzymatic activity of HDAC3 is dependent on its interaction with the conserved deacetylase-activating domain of NCoR or SMRT and targets both acetylated histones and transcription regulators [63,64,65]. Acetylation and deacetylation of histones contribute to the regulation of gene transcription [66,67]. Consistent with this, the inhibitory activity of NCoR/SMRT-HDAC3 complexes recruited by Rev-erbα has been linked to the deacetylation of histones, which prevents chromatin looping between enhancers and promoters and gene activation by the eviction of histone acetylation readers [68]. NCoR/SMRT-HDAC3 complexes also inhibit differentiation of rhabdomyosarcoma cells in an HDAC3 activity-dependent manner that involves the deacetylation of histone 3 K9 [69]. However, repression by NCoR/SMRT does not require HDAC3′s deacetylase activity in some models [70,71,72]. Interestingly, in skin keratinocytes, where papillomavirus replication takes place, NCoR/SMRT-HDAC3 complexes are required in a deacetylase-independent manner for the stepwise epithelial stratification [73].

NCoR/SMRT complexes are recruited by HPV E8^E2 into replication foci induced by overexpression of E1 and E2 [39]. Co-immunoprecipitation analyses indicated that NCoR/SMRT binding is dependent upon the E8 part, and siRNA depletion confirmed that NCoR/SMRT complexes mediate the repression activity of different E8^E2 proteins. While repression activity is dependent upon NCoR/SMRT, genetic depletion of only HDAC3, the only known enzyme in NCoR/SMRT complexes, did surprisingly not interfere with the repression activity of HPV31 E8^E2 [39,49]. However, this study also indicated that the recruitment of E8^E2 reduces RNA polymerase II occupancy at the HR-HPV major early promoter [49], which points to the possibility that the inhibition of transcription might be due to reduced formation of the pre-initiation complex. SMRT was confirmed to be recruited in an E8^E2-dependent manner into HPV16 replication foci in keratinocytes maintaining replicating viral genomes [74]. This study revealed also that the histone variant macroH2A1 is recruited into replication foci by E8^E2 [74]. However, depletion experiments indicated that macroH2A1 does not act as a repressor of HPV16 transcription and thus does most likely not contribute to E8^E2 repression functions [74]. The analysis of histone modifications found no evidence for repressive histone 3 (H3) K9 me2/3 or H3K27me3 marks in HPV31 wt-positive replication foci [74]. However, whereas in HPV16, wt foci RNA polymerase II phospho-S5, a marker for active transcription, was only present on the surface of replication foci, this marker and histone H3K9/K18ac were present throughout the foci in E8^E2 ko cells [74]. This supports the model that E8^E2 interferes with an active pre-initiation complex but also points to the exciting possibility that E8^E2 also influences the functional organization of replication foci.

### 3.4. Modulation of E8^E2 Activity by Phosphorylation

E2 proteins have been shown to be phosphorylated, and this alters their activities [75,76,77,78]. A comparative study using HPV31 E2 and E8^E2 proteins revealed that S78, S81, and S100 in E8^E2 and S266 and S269 in E2 are phosphorylated [79]. Remarkably, S78 turned out to be the major phosphorylation site in E8^E2, whereas the corresponding S266 in E2 was not [79], indicating that the N-termini influence phosphorylation. Functional assays revealed that mutation of S78 changed repression activity of E8^E2, whereas mutation of S266 did not alter E2 transcription activation [79]. Surprisingly, mutation of E8^E2 S78/E2 S266 in HPV31 genomes did not reveal a phosphorylation-dependent regulation of viral genome replication or gene expression in undifferentiated or differentiated cells [79]. However, global transcriptome analyses provided evidence that E8^E2 regulates in a phospho-S78-dependent manner expression of the cellular LYPD2 gene [79]. The function of LYPD2 is currently unknown, but expression has been shown to be higher in cervical cancer tissue compared to surrounding normal tissues [80], supporting the idea that LYPD2 is an HPV-regulated host gene. This suggests that phosphorylated forms of HPV31 E8^E2 might specifically regulate the expression of a subset of host genes.

## 4. Conclusions

The available evidence strongly suggests that E8^E2 proteins are highly conserved repressors of PV replication. Despite the conserved short-term phenotype of increased viral genome replication and gene expression, long-term phenotypes of E8^E2 knock outs of different human and animal PV in tissue culture and animal models vary considerably. The reason for this remains obscure, but the different requirements for E8^E2 could be related to virus-type dependent activities of other viral proteins or different post-translational modifications of E8^E2 proteins.

## Data Availability

Not applicable.
